# Moiré Modulation of Van Der Waals Potential in Twisted Hexagonal Boron Nitride

**DOI:** 10.1021/acsnano.1c11107

**Published:** 2022-04-29

**Authors:** Stefano Chiodini, James Kerfoot, Giacomo Venturi, Sandro Mignuzzi, Evgeny M. Alexeev, Bárbara Teixeira Rosa, Sefaattin Tongay, Takashi Taniguchi, Kenji Watanabe, Andrea C. Ferrari, Antonio Ambrosio

**Affiliations:** † Center for Nano Science and Technology, Fondazione Istituto Italiano di Tecnologia, Via G. Pascoli 70, Milan 20133, Italy; ‡ Cambridge Graphene Centre, University of Cambridge, 9, JJ Thomson Avenue, Cambridge CB3 0FA, United Kingdom; § Physics Department, Politecnico Milano, P.zza Leonardo Da Vinci 32, Milan 20133, Italy; ∥ School for Engineering of Matter, Transport and Energy, Arizona State University, Tempe, Arizona 85287, United States; ⊥ International Center for Materials Nanoarchitectonics, National Institute for Materials Science, 1-1 Namiki, Tsukuba 305-0044, Japan; # Research Center for Functional Materials, National Institute for Materials Science, 1-1 Namiki, Tsukuba 305-0044, Japan

**Keywords:** layered materials, moiré superlattices, hexagonal boron nitride, atomic force microscopy, van der Waals interactions, mechanical phase imaging

## Abstract

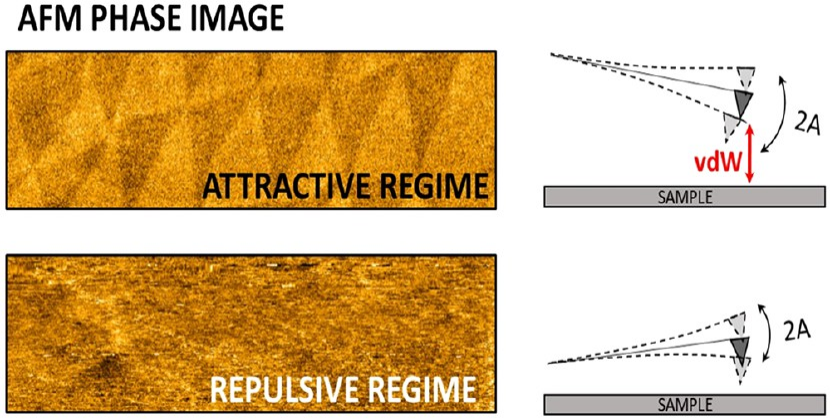

When a twist angle is applied between two layered materials (LMs), the registry of the layers and the associated change in their functional properties are spatially modulated, and a moiré superlattice arises. Several works explored the optical, electric, and electromechanical moiré-dependent properties of such twisted LMs but, to the best of our knowledge, no direct visualization and quantification of van der Waals (vdW) interlayer interactions has been presented, so far. Here, we use tapping mode atomic force microscopy phase-imaging to probe the spatial modulation of the vdW potential in twisted hexagonal boron nitride. We find a moiré superlattice in the phase channel only when noncontact (long-range) forces are probed, revealing the modulation of the vdW potential at the sample surface, following AB and BA stacking domains. The creation of scalable electrostatic domains, modulating the vdW potential at the interface with the environment by means of layer twisting, could be used for local adhesion engineering and surface functionalization by affecting the deposition of molecules or nanoparticles.

Layered materials (LMs) are promising both for device applications and for the exploration of fundamental physics.^1^ In graphene and related materials (GRMs), such as hexagonal boron nitride (hBN) and transition metal dichalcogenides (TMDs), each layer is bonded by covalent in-plane bonds, whereas weaker van der Waals (vdW) forces hold the layers together.^1^ The LM properties can be tuned by controlling the twist angle between layers, producing a spatially modulated interlayer registry, known as moiré superlattice.^2−4^ This can lead to superconductivity^5^ and Mott-like insulator states^6^ in twisted graphene bilayers, long-lived interlayer excitonic states in monolayer (1L) MoSe_2_/WSe_2_ heterostructures,^7^ and resonant tunneling of graphene Dirac Fermions.^8,9^

hBN is a wide-bandgap (∼6 eV)^10^ insulating LM with a peculiar set of optical,^11−17^ mechanical,^18,19^ and electrical properties.^20−22^ It is commonly used as an encapsulating material in GRMs.^23^ It also gained interest in the context of moiré physics. For example, scattering near-field optical microscopy (s-SNOM) uncovered the variation of the in-plane optical phonon frequencies for different stacking in the moiré superlattice of a twisted hBN (t-hBN).^24^ Piezo force microscopy revealed strain gradients along moiré stacking domain boundaries, through piezoelectric coupling to an electric field applied between atomic force microscope (AFM) tip and hBN sample.^19^ Electrostatic force microscopy (EFM) and kelvin probe force microscopy (KPFM) were performed on t-hBN (1–20L-BN on top of a thicker >30L flake^20^), addressing the existence of two opposite permanent out-of-plane polarizations emerging from the moiré pattern.^20−22^ However, the impact of moiré superlattices on local vdW interactions in twisted LMs has not been explored so far, to the best of our knowledge.

Here, we investigate the moiré interlayer modulation of the vdW potential of t-hBN by using tapping mode AFM phase-imaging, a widely used tool for nanoscale force characterization.^25^ In tapping mode AFM, the sine of the phase channel is proportional to the energy dissipated in the tip–sample interaction.^26−33^ This depends on the tip–sample distance in a way that is specific to the probed force,^26^ allowing noncontact (or long-range) vdW forces to be distinguished from other local interactions, such as capillary, surface energy hysteresis, and viscoelasticity forces.^26,34^ By tuning the phase channel to the local vdW dissipation, we quantify the dissipated energy and visualize the modulated vdW potential at the top layer–air interface, resulting from the t-hBN moiré superlattices. We provide a physical interpretation of the nanoscale origin of the vdW dissipation contrast based on analysis of the tip–sample interaction, showing that the Debye force between the neutral tip and interlayer permanent electric dipoles is the principal source of the imaging contrast. We explain this Debye interaction for the two main stacking domains involved in the t-hBN structure, i.e., AB and BA.

AFM phase imaging is a simpler and more reliable way to visualize moiré patterns in t-LMs. Unlike electric force microscopy techniques, such as EFM and KPFM, it does not require any specific sample or tip biasing. This simplifies sample preparation and reduces the possibility of damage.

Weak electrostatic potentials at the interface with the environment are at the origin of numerous phenomena in fields ranging from fluid dynamics^35^ to tribology,^36^ both at the macroscopic and microscopic level.^36^ We find a modulation on the vdW potential at the sample surface in t-hBN and quantify the related energy dissipation, after calibration of the AFM parameters. The fact that such potential can be patterned in scalable domains engineered by twisting provides a tool for functionalization of surfaces. Locally engineered adhesion, periodically spaced anchoring sites for molecules and nanoparticles deposition, and electrostatically patterned substrates for controlled cells stimulation are a few applications that could benefit from our findings.

## Results and Discussion

**Figure 1:**
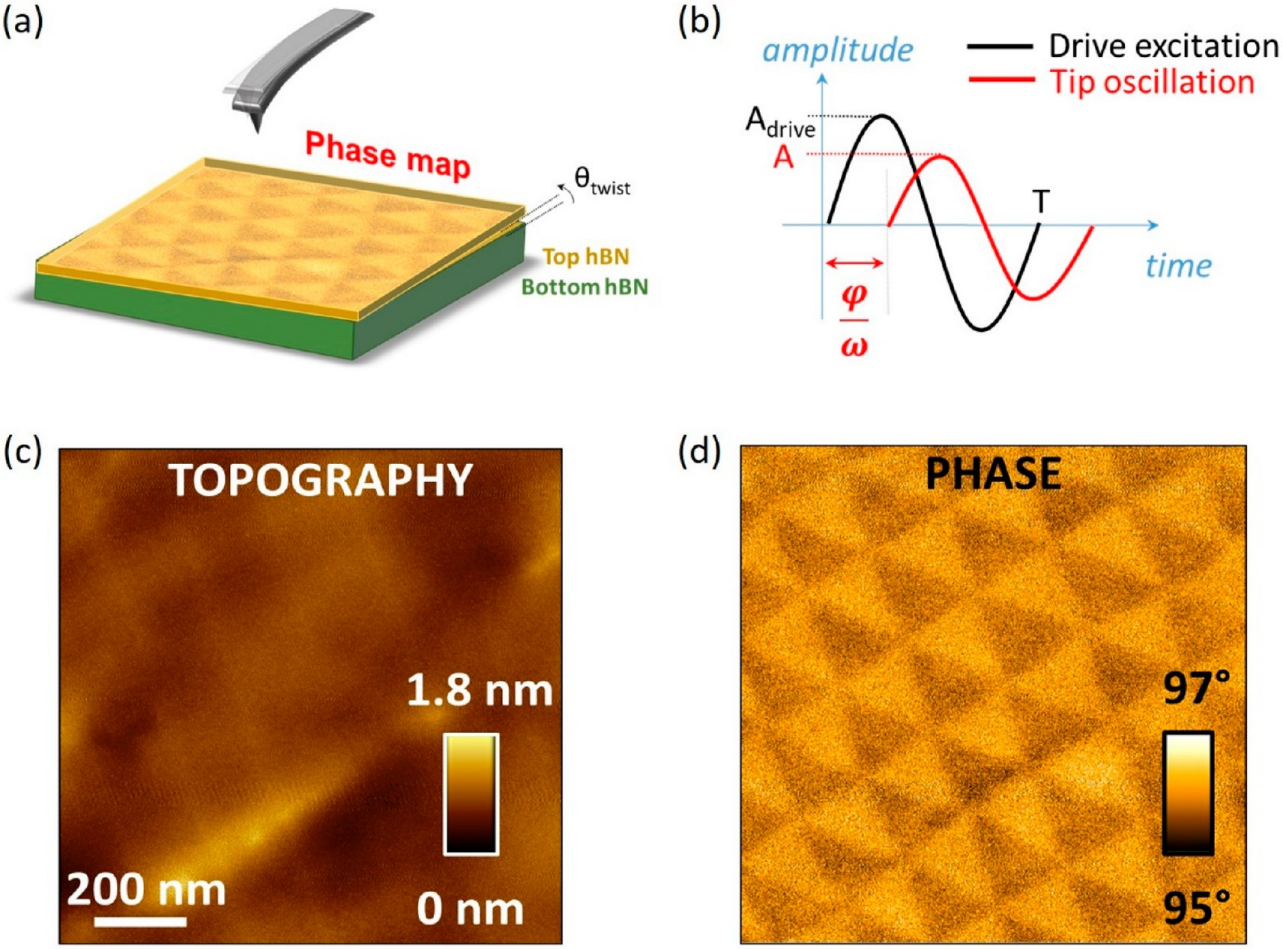
Tapping mode AFM imaging of t-hBN. (a) Schematic of t-hBN (2 nm/8 nm, θ_twist_ ∼ 0°) sample. (b) Plot of the two main signals involved in AFM phase-imaging, i.e., drive excitation (black) and tip oscillation response (red). *A*_drive_ and *A* are reported, together φ, *T* = 2π/ω, ω = 2*πν* (*v* is the cantilever first resonance frequency). (c) Representative AFM topography of top hBN, showing a flat morphology. (d) Corresponding AFM (attractive) phase channel where the moiré superlattice is visible. Imaging parameters for (c,d): *A*_0_ = 5.3 nm, *A* = 5.1 nm, free phase ∼86°. Cantilever: Scanasyst fluid (Bruker, *k* ∼ 0.7 N·m^–1^).

We use a t-hBN sample consisting of a 2 nm (∼5L) top hBN layer and an 8 nm (∼20L) bottom hBN, Figure 1a, on Si + 285 nm SiO_2_, as described in Methods. The twist angle, θ_twist_, is defined as the angle between the lattice vectors of the top and bottom hBN flakes.^37,38^ We control this by first identifying neighboring flakes cleaved from the same bulk hBN, as determined by inspection of the relative orientation of their faceted edges, and then picking one flake up using the other.^37^ θ_twist_ may be tailored by rotating the transfer stage between picking up the first and second flakes. The accuracy of θ_twist_ is limited by the resolution and wobble of the transfer stage (±0.01° and ±0.008°), monitored by tracking the relative orientations of the faceted edges of top and bottom hBN using optical microscopy and AFM.

AFM/phase/KPFM measurements are taken at ∼25 °C (RH ∼ 40%), using a Multimode 8 (Bruker) AFM microscope, with Scanasyst Fluid (Bruker, *k* ∼ 0.7 N·m^–1^, *v* ∼ 150 kHz), Scanasyst Air HR (Bruker, *k* ∼ 0.4 N·m^–1^, *v* ∼ 130 kHz), 240AC-NG (OPUS, *k* ∼ 2 N·m^–1^, *v* ∼ 70 kHz), and ASYELEC.01-R2 (Asylum Research, *k* ∼ 2.8 N·m^–1^, *v* ∼ 75 kHz) cantilevers. To avoid damaging the tips, calibration procedures are performed at the end of the experiments. The deflection sensitivity is obtained by recording 10 force–distance curves on mica (without changing the laser spot position onto the cantilever) and calculating the average inverse slope of the contact region. The cantilevers spring constant is then obtained using the standard thermal tune method.^39^ All the AFM images are obtained in tapping mode at ∼0.5–1 Hz scan rate. These are all postprocessed using Gwyddion.^40^ Phase imaging theory^25^ states that phase contrast is inversely related to the cantilever spring constant. This points to the need of a soft cantilever. Thus, phase images are taken with *k* ∼ 1 N·m^–1^. No phase moiré contrast is obtained for *k* ∼ 30 N·m^–1^ (Cantilever: PPP-NCHAuD, Nanosensors). KPFM maps are also taken with soft cantilevers with the sample holder connected to ground.

AFM phase values tend to follow different conventions depending on the AFM microscope brand. Ref (41) summarized all of them. Bruker’s microscopes usually set the free phase (i.e., the phase delay between tip oscillation and cantilever excitation when the cantilever is far from the sample^25^) to 0°, forcing the attractive regime (AR) to correspond to negative phase values and the repulsive regime (RR) to positive ones. Instead, Asylum Research AFM microscopes set the free phase to 90°, with AR (RR) phase values higher (lower) than this. AFM tapping mode force spectroscopy can be performed to verify these definitions. All phase values in the rest of this paper are renormalized from Bruker to Asylum Research convention.

We simultaneously record topography and phase channels in tapping mode AFM. The phase signal can be described in terms of a forced and damped harmonic oscillator model^42^ applied to the dynamics of the AFM cantilever. Figure 1b shows the main parameters. If the cantilever driving excitation is represented by a harmonic signal, i.e., *A*_drive_ sin(*ωt*) (with *A*_drive_ the drive oscillation amplitude, ω = 2*πν*, with *v* the cantilever first resonance frequency, and *t* the time), the tip oscillation corresponds to a delayed sinusoidal motion, i.e., *A* sin(ω*t* – φ) (with *A* the tip oscillation amplitude, kept constant to a set-point by the feedback electronics, and φ the phase signal). *A*_drive_ is related to the free oscillation amplitude *A*_0_ (i.e., the tip oscillation amplitude when the tip is hundreds nm from the sample) as , with *Q* the quality factor of the cantilever resonance.^42^

Figure 1c is a representative tapping mode AFM topography image of the air/hBN interface. The morphology is flat, with 1.8 nm modulation over a 1 μm × 1 μm scanned area. The corresponding phase image in Figure 1d, instead, has a periodic pattern characterized by triangular domains with a typical dimension ∼200 nm, consistent with previous observations of moiré superlattices with electrical AFM modes.^20−22^ In Methods we provide a direct comparison between our approach and KPFM.

**Figure 2:**
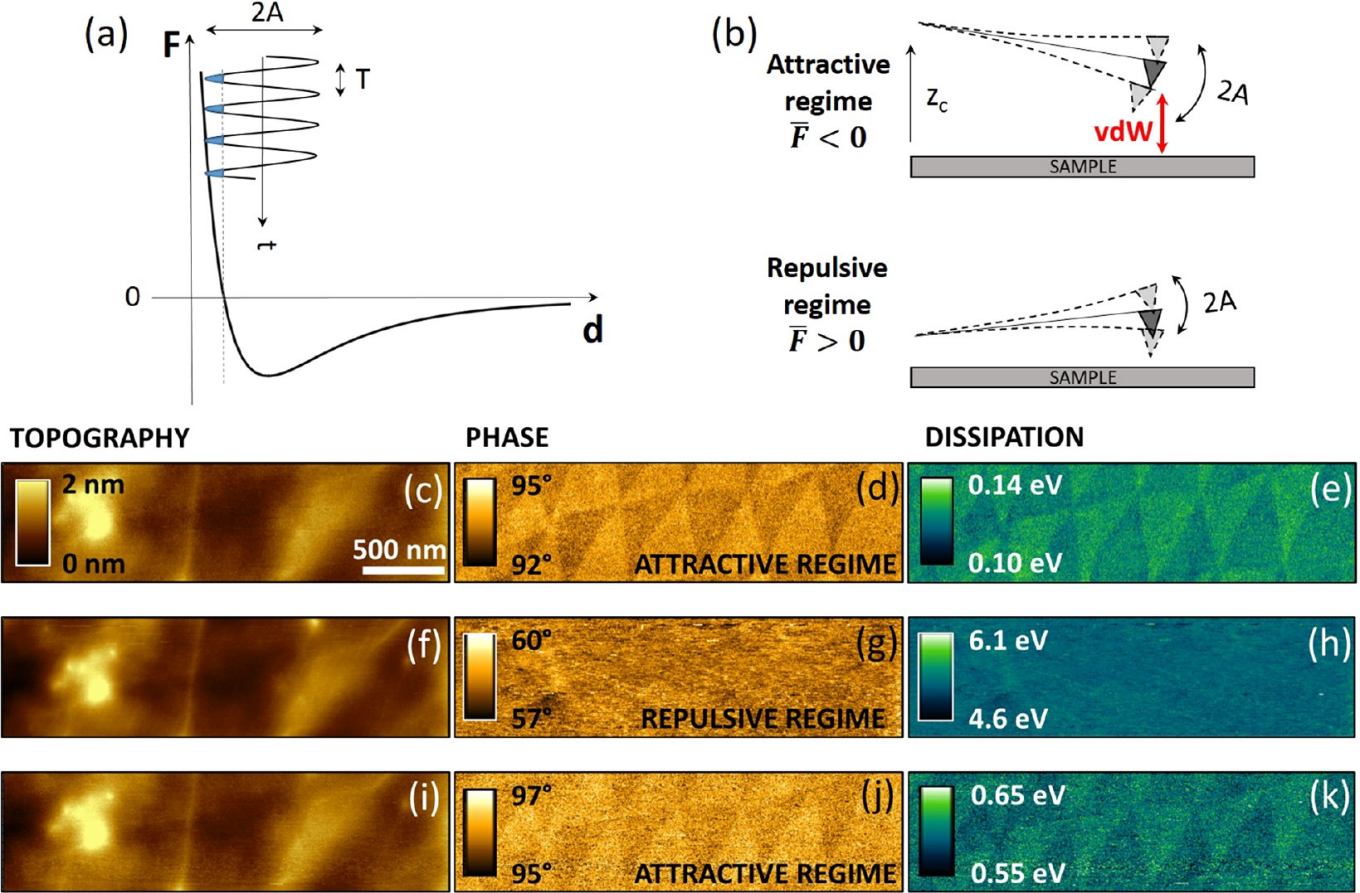
Force regime-dependent tapping mode AFM imaging of moiré contrast in t-hBN. (a) Lennard-Jones (LJ) force–distance (*F*–*d*) plot showing a region of negative (attractive) and positive (repulsive) interaction between tip and sample. The AFM tip oscillation is reported onto the LJ graph, addressing both attractive and repulsive (shaded in blue) interactions inside each single oscillation period *T*. (b) Schematic of the vdW-related AR (for *z*_*c*_ ≤ *A*_0_) characterized by a negative *F̅* and cantilever deflection, and RR (for *z*_*c*_ ≪ *A*_0_) where, instead, *F̅* and deflection are positive. The average cantilever position is shaded in dark gray. (c–e) Topography of a specific top hBN area and its corresponding phase and dissipation maps once imaged in AR (φ > 90°). The moiré pattern is visible. (f–h) Same channels as in (c–e), in RR (φ < 90°), where the moiré contrast is lost. (i–k) Topography, phase and dissipation once the AR (and the moiré superlattice map) is restored. Imaging parameters for c–m: *Q* = 135, *A*_0_ = 18.7 nm, *A* (AR) = 18.3 nm and *A* (RR) = 11.8 nm. Cantilever: Scanasyst Air HR (Bruker, *k* = 0.75 N·m^–1^).

Figure 1d is obtained when the tapping mode probe is operated in AR.^25^ The AR and RR concepts in AFM phase-imaging can be described in terms of the nonlinear AFM cantilever dynamics.^43^ Assuming the tip–sample interaction to be described by a Lennard-Jones force curve, the tip experiences, over the oscillation period *T*, either attractive (force *F* < 0) or repulsive (*F* > 0) interactions, depending on the instantaneous tip–sample distance (see Figure 2a, where oscillation regions characterized by repulsive forces are shaded in light blue).

An average force, *F̅*, can be calculated as the integral of the instantaneous force over one tip oscillation period: *F̅* = 1/*T* ∫_0_^*T*^*F*(*t*) d*t*. Approaching the tip to the sample, i.e., decreasing the distance *z*_*c*_ of the cantilever chip from the sample (Figure 2b), two probing regimes can be defined: 1) AR, when the tip is far from the sample (*z*_*c*_ ≤ *A*_0_, *F̅* < 0); 2) RR, for *z*_*c*_ ≪ *A*_0_, *F̅* > 0. Thus, in AR (RR) the AFM cantilever experiences an average negative (positive) deflection, Figure 2b. The AFM phase channel is a useful tool for monitoring/tuning these two probing regimes. AR and RR correspond to phase values φ > 90° and φ < 90°, respectively.^43^ It is possible to move from one to the other by modifying *A*_0_ and *A*. In our case, to visualize the moiré superlattice via the phase channel, it is necessary to operate in AR.

Figure 2c–j plot topography and phase images of the same hBN region in AR and RR. The topography does not provide any contrast related to the moiré superlattice in any of the operating regimes. The phase images instead show a pattern of triangular domains only in AR. When the oscillation regime is switched from AR to RR (from φ > 90° to φ < 90°), decreasing *A* while keeping constant *A*_0_, the topography is unaltered (Figure 2c,f), while the moiré contrast completely disappears in the phase map (Figure 2d,g). The moiré pattern is recovered by restoring the AR imaging parameters (Figure 2j).

To test the general applicability of our methodology, we image moiré superlattices in different regions of the same sample, with different cantilevers (spring constant *k* ∼ 1 N·m^–1^) and scan size, and on a different t-hBN; see Methods.

Further insights can be obtained by introducing the local dissipation energy of the tip–sample interaction. As discussed in refs (26−32, 44, 45), this can be retrieved through the phase shift between drive excitation and tip oscillation:^46,47^ sin φ is proportional to the energy of the tip–sample dissipative interaction. For a sinusoidal oscillation of a cantilever driven at its resonance frequency, the dissipated energy *E*_diss_ (in one tip oscillation *T*) and sin φ are linked by:^26^
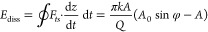
1*F*_*ts*_ is the total tip–sample interaction and d*z*/d*t* the tip speed along the *z*-axis at time *t*. Eq 1 can be considered accurate as long as the dissipative phenomenon does not take place in a low-*Q*(<10) environment.^31^ In this case, contributions from higher cantilever modes should be considered and a sinusoidal oscillation cannot be accepted.^31^ Our *Q* ∼ 150 (derived by fitting the resonance curve)^25^ allows us to use eq 1.

Once a phase image is acquired, a dissipation map can be then reconstructed through eq 1, provided a calibration of *k*, *Q*, *A*, and *A*_0_ is done. Figure 2e,h,k are dissipation energy maps obtained from the phase maps of Figure 2d,g,j by applying eq 1. As for the dissipation maps, the triangular domains of the moiré superlattice are evident only while operating in AR.

**Figure 3:**
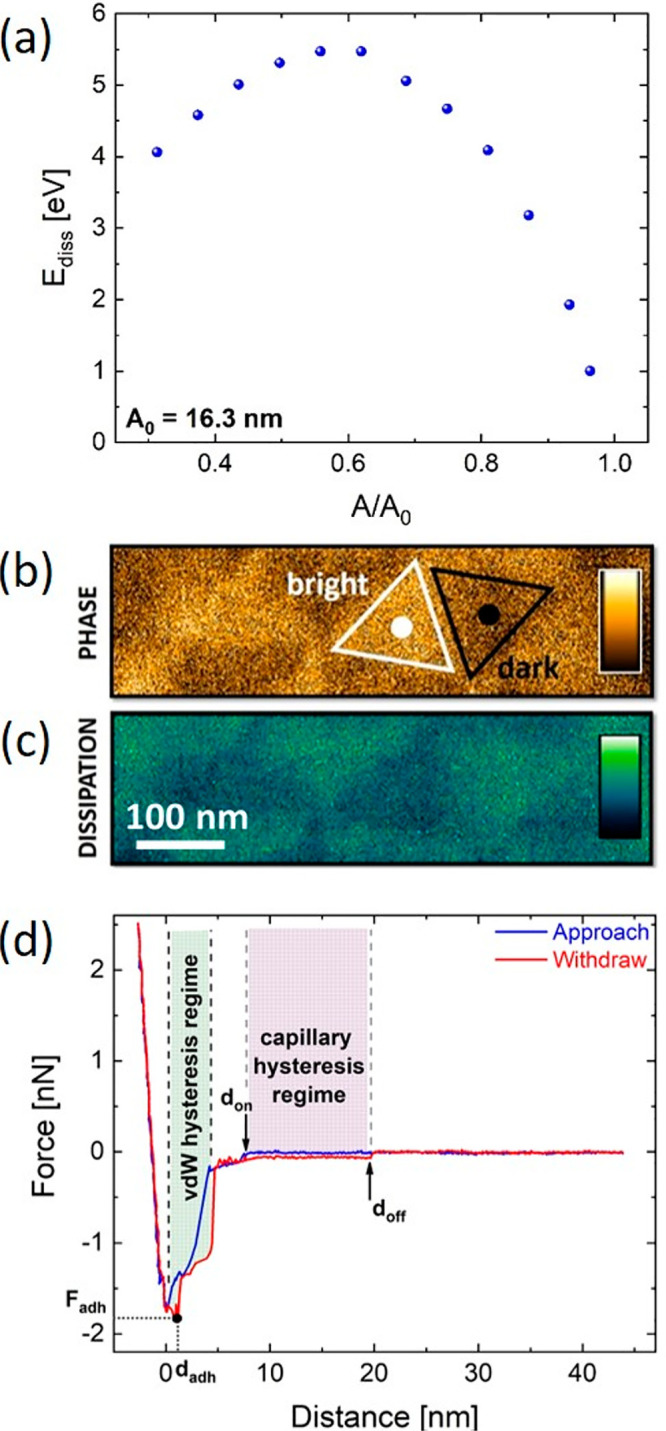
(a) Dissipation energy vs *A*/*A*_0_ from all data in Methods. The peak indicates a long-range vdW origin of tip–sample dissipation. (b) AFM phase image revealing bright and dark triangular moiré phase domains. The white and black dots show the position where *n* = 300 AFM force–distance curves are taken (ramp speed= 100 nm/s, ramp distance = 50 nm). Color scale: from 101° to 104°. (c) Dissipation map corresponding to (b), via eq 1. Color scale: from 4.7 to 6.4 eV. (d) Representative force–distance curve on an AB domain. *d*_on_ and *d*_off_ are the distance of formation and rupture of the nanoscale water bridge between tip and sample. The shaded regions highlight the two hysteresis regimes where approach and withdraw curves do not overlap. The adhesion force *F*_adh_ (i.e., the minimum force of the withdraw curve) is also marked. Imaging parameters for (a) are given in Methods. Cantilever for (d): Scanasyst Air HR (Bruker, *k* = 0.75 N·m^–1^).

Dissipation in AFM measurements can have different origins.^27^ In terms of local interactions at the nanoscale, 3 main dissipative mechanisms at the tip–sample junction can be considered:^26^ (1) long-range (i.e., no tip–sample mechanical contact) vdW-like forces; (2) short-range surface energy hysteresis; and (3) short-range viscoelasticity. Ultimately, all are characterized by a different force expression when the tip approaches (forward movement) or withdraws (backward movement) to/from the sample surface, resulting in what is usually called a force–distance hysteretic behavior.^48^ These differ for their dependence on the minimum tip–sample distance, *d*_min_, that can be controlled by adjusting *A*/*A*_0__._^26,28^ Plotting the dissipation energy as a function of *A*/*A*_0_ enables the identification of the main dissipation channel responsible for the moiré superlattice contrast of Figure 1d. In practice, the same t-hBN area is scanned several (∼10) times (with no appreciable drift of the image upon consecutive scanning), keeping *A*_0_ constant and decreasing, at each image, *A*; see Methods for details. Figure 3a plots *E*_diss_ as a function of *A*/*A*_0_. The trend in Figure 3a is typical of long-range vdW forces.^26,28^ Since surface energy hysteresis and viscoelasticity emerge from a tip–sample interaction typical of the repulsive regime, we consider them negligible in the operating attractive regime.

Even though the energy dissipation trend excludes short-range forces as the moiré imaging contrast mechanism, besides vdW forces, capillary forces could, in-principle, contribute to a similar dissipation behavior.^34^ In this case, the contribution of capillary forces would result from the presence of an uncontrolled water layer on the sample, due to ambient humidity (all our AFM measurements are in air at RH ∼ 40%).

In order to distinguish between capillary and vdW forces as dissipative mechanisms, we perform AFM force-spectroscopy. In this case, the tip is not oscillated, but approached and withdrawn to/from the sample, while recording the deflection of the cantilever. *n* = 300 force–distance curves are collected at the center of both “dark” and “bright” domains of a previously acquired phase map, Figure 3b. Hooke’s law allows the force on the tip to be quantified by multiplying the measured cantilever deflection by *k*.

As discussed in Methods, the comparison of phase and KPFM maps on the same region allows the identification of bright (dark) phase domains as AB (BA) stacking domains. We use this domain classification in the following.

Figure 3d plots a representative *F*–*d* curve for an AB domain (analogous considerations hold for BA domains as reported in Methods). The blue and red curves show different features. While approaching the tip to the sample surface (blue), a zero-force condition can be found for *d* > *d*_on_, for which tip and sample are far enough that any interaction is negligible. Then, as the tip moves closer to the surface, a small step in the force appears (for *d = d*_on_) that can be ascribed to the formation of a capillary bridge between tip and sample.^49−51^ At such distances, attractive vdW forces can affect the tip–sample interaction.^48^

When the gradient of the total attractive force overcomes the spring constant of the cantilever, a sudden collapse of the tip toward the sample takes place, caused by the so-called snap-in mechanical instability.^48^ At this point, the tip contacts the sample entering a repulsive regime, with a force increasing until set-point deflection is reached.

Retracting the tip from the sample (red), in RR, two separated regions can be distinguished, where approach and withdraw curves are not overlapping, signature of hysteresis. In such regions, the dissipation can be calculated as the enclosed area between the approach and withdraw curves. One hysteresis region extends from *d*_on_ to *d*_off_, where *d*_off_ corresponds to the distance of rupture of the capillary bridge;^49−51^ the other from the adhesion point (*d*_adh_, *F*_adh_) to the end of the snap-out^48^ (i.e., the equivalent of the snap-in, but for the retraction curve). In this region, the nanoscale water bridge is not broken yet (since *d* < *d*_off_); therefore, any dissipative contribution only results from vdW forces; see Methods for further details.

This allows us to distinguish the contribution of vdW forces from capillary ones. In our measurements, when the tip is oscillating, *A*_0_ is ∼16 nm, Figure 3a. In this case, a maximum tip oscillation ∼32 nm (= 2·*A*_0_) is spanned, covering both vdW and capillary interaction regions. These results, for both AB and BA stacking (see Methods), restrict the capillary contribution to ∼20% of the total dissipation. Thus, the origin of the contrast in the moiré patterns in the phase map of Figure 1d is mainly due to a modulation of the interlayer vdW potential in the moiré superlattice. The extension of the vdW dissipation regime is restricted to the first five-to-ten nm above the top hBN surface, Figure 3d.

VdW forces emerge from the quantum mechanical interaction between permanent or transient electric dipoles between molecules,^52^ i.e., the AFM tip apex and the forefront sample atoms. Casimir forces^53^ can be ruled out since they are usually detected on a much larger atoms ensemble by using μm radius spheres rather than sharp tips.^54^ Thus, 3 vdW interaction classes can be considered:^52^ London, Debye, and Keesom. London forces^55^ are the consequence of the interaction between two neutral molecules, whose quantum temporary dipole moments come to a close distance (tens of nm). Debye forces^56^ affect a neutral molecule interacting with a polar molecule. Keesom forces^57^ emerge from the interaction between two polar molecules. All have an attractive energy *U*_vdW_ ∝ 1/*d*^6^, where *d* is the distance between the two parts.^52^

Refs (20−22) suggested that a layer of ferroelectric dipoles is present at the interface between top and bottom hBN, due to the marginal (<1°) θ_twist_ between the two crystal structures. Hence, our moiré phase-image contrast emerges from the Debye dissipative vdW interaction between tip and sample.

The values of dissipation energy related only to the vdW contribution (*E*_diss_^vdW^) are in Table 1 for AB and BA domains. These show higher average vdW dissipation energy for BA than AB. This can be qualitatively explained in terms of the different Debye interaction between tip and AB or BA domains. While AB and BA stacking domains both have out-of-plane electric dipole moment densities, the positions of their effective dipole centers of mass along the direction orthogonal to the layers are different (Figure 8e,f). The effective dipole center of mass is closer to the surface for BA, resulting in larger vdW forces acting on the AFM tip.

**Table 1:** *E*_diss_^vdW^ and Adhesion Force from 300 *F*–*d* Curves on the Center of Dark (BA) and Bright (AB) Stacking Domains (Figures 3b, 8a,b)^a^

*n* = 300	*E*_diss_^vdW^ [eV]	adhesion [nN]
Dark (BA)	7.97 ± 0.33	1.764 ± 0.006
Bright (AB)	6.80 ± 0.34	1.749 + 0.004
Δ	1.17 ± 0.47	0.015 + 0.007

a *E*_diss_^vdW^ is obtained calculating the area between approach and withdraw *F*–*d* curves restricted to the vdW hysteresis regime. Δ is the difference between dark (BA) and bright (AB) vdW dissipation energies.

t-hBN is not the only LM expected to have vertical polarization domains.^58^ Indeed, we observe moiré domains via AFM phase imaging in t-WSe_2_; see Figure 16. This shows the general applicability of our imaging approach for LMs.

## Conclusions

We observed the spatial modulation of the vdW potential induced by the moiré superlattice of t-hBN and t-WSe_2_, via tapping mode AFM phase-imaging, without sample or tip biasing. Our tapping mode AFM phase-imaging is a noninvasive probe for the visualization of the interlayer vdW potential in moiré superlattices, with no external sample perturbations and compatibility with functional electronic devices in air/liquid/vacuum. By tuning the tip–sample force to the attractive regime, where mainly long-range vdW forces are probed, repulsive interactions were discarded, allowing the visualization of two different triangular vdW domains (AB and BA) emerging from the moiré superlattice. We quantified the vdW interactions on both AB and BA regions, through the proportionality between phase signal and dissipative tip–sample forces, indicating the BA regions as the most dissipative. We discussed the origin of this nanoscale vdW dissipation and related the interaction between tip and interlayer electric dipoles to a Debye vdW force.

The modulation of the electrostatic potential on the samples, the domain extension and their size can be engineered by twisting the layers. This provides a tool in surface functionalization, enabling to locally tune the electrostatic interaction with the environment on a large scale (>1000 μm^2^),^19,59^ while maintaining a nm resolution. Nanopatterning is an important and diverse research topic continuously enriched by different approaches.^60^ Of particular relevance is high-spatial resolution combined with large scale patterning (see, e.g., refs (59−61)). LM twisting results in nanopatterning of the interlayer bonding, with periodical domains whose size is tuned by the twist angle.^62^ Our results indicate that the twist also results into nanopatterning of the electrostatic field at the sample/environment interface. The modulation produces a local field nanopatterning with the periodicity and tunability of the moiré pattern. We can then foresee that moiré superlattices in insulating and semiconducting LMs could complement already known patterning techniques by lifting the requirement for any sample pretreatment, as for chemical-assisted patterning,^60^ or the need for external fields, as in field-assisted patterning.^60^

## Methods

### Sample Preparation and Raman Characterization

t-hBN samples are prepared by first exfoliating bulk hBN (B-hBN) crystals, grown at high pressure and temperature in a barium boron nitride solvent,^63^ onto Si + 90 nm SiO_2_ by micromechanical cleavage (MC). In order to control θ_twist_, either large flakes (>50 μm) selectively torn during transfer^37^ or neighboring hBN flakes cleaved from the same bulk crystal during MC^20^ are identified by studying the orientation of their faceted edges using optical microscopy.^64^ t-hBN samples with controlled *inter*layer rotation are then fabricated using polycarbonate (PC) stamps.^65^ First, a PC film on polydimethylsiloxane (PDMS) is brought into contact with the substrate with hBN flakes at 40 °C using a micromanipulator, so that the contact front between stamp and substrate covers part of one flake or one of two adjacent flakes exfoliated from the same crystal on the tape. Stamps are then retracted, and the material in contact with the PC is picked up from the substrate. After picking up the first flake, a controlled θ_twist_ (±0.01°, as determined by the resolution and wobble of the rotation stage) can be applied by rotating the sample stage, before the flake on PC is aligned to the second one and brought into contact at 40 °C. The stamp is then retracted and the resulting t-hBN is picked up by PC. t-hBN is then transferred onto Si + 285 nm SiO_2_ at 180 °C, before the PC residue is removed by immersion in chloroform and then ethanol for 30 min. While Si + 90 nm SiO_2_ is used to facilitate the identification of hBN flakes,^66^ Si + 285 nm SiO_2_ is chosen for further characterization, such as gate dependent electrical measurements.

Ultralow frequency (ULF) Raman spectroscopy may be used in order to estimate the number of layers, *N*, of hBN by measuring the position of the C mode,^67,68^ Pos(C). For *N* > 5, the shift in Pos(C), ΔPos(C), can be smaller than the spectral resolution (e.g., ΔPos(C) ∼ 0.15 cm^–1^ between *N* = 10 and *N* = 11 vs a resolution of ∼0.6 cm^–1^, corresponding to the wavenumber interval between detector pixels for the combination of diffraction grating and CCD used in the measurements). However, as the Raman peaks are represented by multiple data points even for spectrally narrow ULF modes (e.g., >5 data points for the C mode), it is possible to extract their position with accuracy exceeding the spectral resolution of the experimental setup, via spectral fitting. In general, the error of the peak position extracted via fitting is determined by the fitting error, statistical errors arising from spatial variation, CCD noise and errors associated with the registry of pixels relative to the position of peaks.

**Figure 4:**
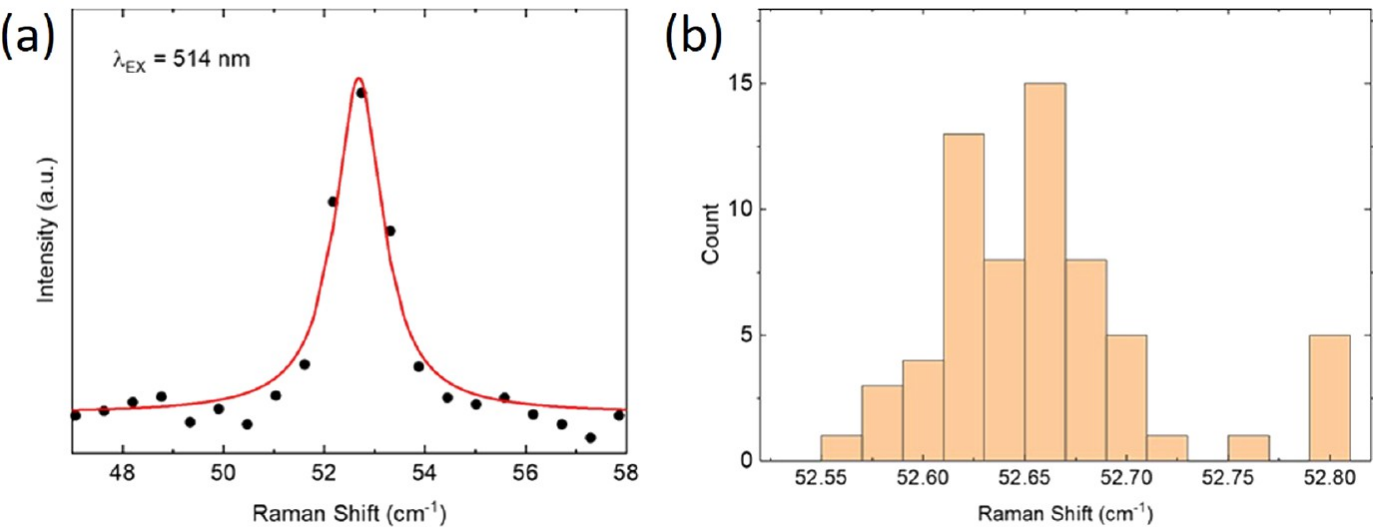
(a) C peak of *N* > 15 l-hBN on Si + 90 nm SiO_2_, with a Lorentzian fit. (b) Distribution of Pos(C) from 64 separate measurements across the same hBN flake.

In order to extract the error of our measurements for Pos(C) due to fitting and statistical variations, a series of ULF Raman spectra are measured on *N* > 15 l-hBN using a Horiba LabRAM Evolution at 514 nm, with an 1800 l/mm grating and volume Bragg filters with a ∼5 cm^–1^ cutoff frequency and a 100× objective (NA: 0.9). Figure 4a shows good agreement between fit and experimental data. The error associated with the Lorentzian fitting is ∼0.03 cm^–1^, expected to be negligible compared to statistical errors and pixel registry. In order to evaluate the error due to detector noise, lateral variations across the sample surface and other statistical variations, a series of spectra are acquired at different positions on the same hBN flake. A histogram of Pos(C), from 64 different locations is shown in Figure 4b. The mean Pos(C) is ∼52.67 cm^–1^, with a standard deviation ∼0.05 cm^–1^ and a variation range ∼0.25 cm^–1^, which compares favorably with the spectral resolution of the system (∼0.6 cm^–1^).

**Figure 5:**
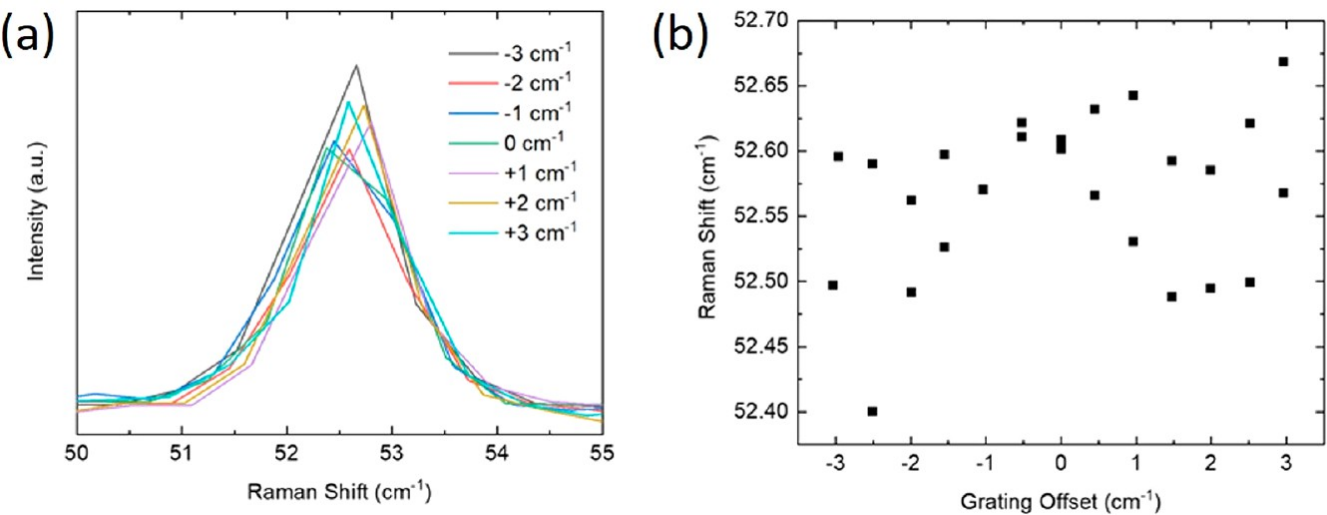
(a) C peak of a *N* > 15 l-hBN flake for different offsets of the spectrometer grating. (b) Pos(C) as a function of grating offset.

As the spectral resolution of the system used is comparable to the full width half-maximum, FWHM(C) ∼ 1.1 cm^–1^, such that the C peak is depicted by <10 pixels, the registry of the CCD pixels is expected to contribute an additional error. To evaluate this, Pos(C) is extracted by fitting spectra acquired from the same position of a *N* > 15 l-hBN flake, with grating position offset from −3 to +3 cm^–1^ in 0.5 cm^–1^ increments, Figure 5.

A range of grating registries are used so that Pos(C) is at the center of two adjacent pixels or between them. The standard deviation of Pos(C), extracted from Lorentzian fitting, is ∼0.06 cm^–1^, with a variation range ∼0.27 cm^–1^, less than the spectral resolution of the system. The values of the main fitting errors are in Table 2.

**Table 2:** Representative Errors in Pos(C) Due to Fitting, Statistical Variation, and Pixel Registry for a *N* > 15 hBN Flake

	fitting error	statistical variation	pixel registry	total error
error (cm^–1^)	±0.03	±0.05	±0.06	±0.14

As the relative change of Pos(C) reduces with increasing *N*,^67,68^ for the hBN flakes used here the change in Pos(C) ∼ 0.15 cm^–1^ between *N* = 10 and 11 is comparable with the total fitting error ∼ ± 0.15, allowing *N* to be determined ±1 layer for *N* < 11.

**Figure 6:**
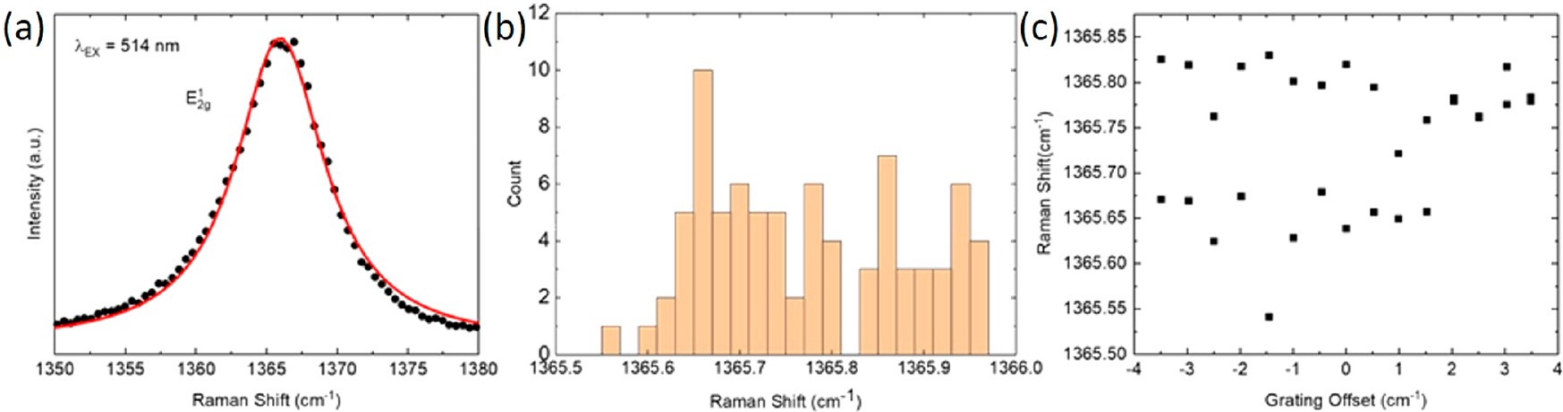
(a) E_2g_ mode in a *N* > 15 l-hBN flake, with Lorentzian fitting. (b) Variation of Pos(E_2g_) for 81 spectra acquired at different positions on the flake. (c) Variation of Pos(E_2g_) for different CCD detection pixel registries, by acquiring spectra at a single position on the sample for different spectrometer grating offsets.

Figure 6 shows the same analysis for the hBN E_2g_ mode ∼1366 cm^–1^.^69−71^ The errors associated with fitting it to a Lorentzian, from statistical variation, and pixel registry are summarized in Table 3. The differences compared to Table 2 are due to an increase in FWHM and intensity (relative to the background) for the E_2g_ mode relative to C.

**Table 3:** Representative Errors in Pos(E_2g_) Due to Fitting, Lateral Statistical Variation, and Pixel Registry for a *N* > 15 hBN Flake

	fitting error	statistical variation	pixel registry	total error
error (cm^–1^)	±0.01	±0.11	±0.08	±0.20

**Figure 7:**
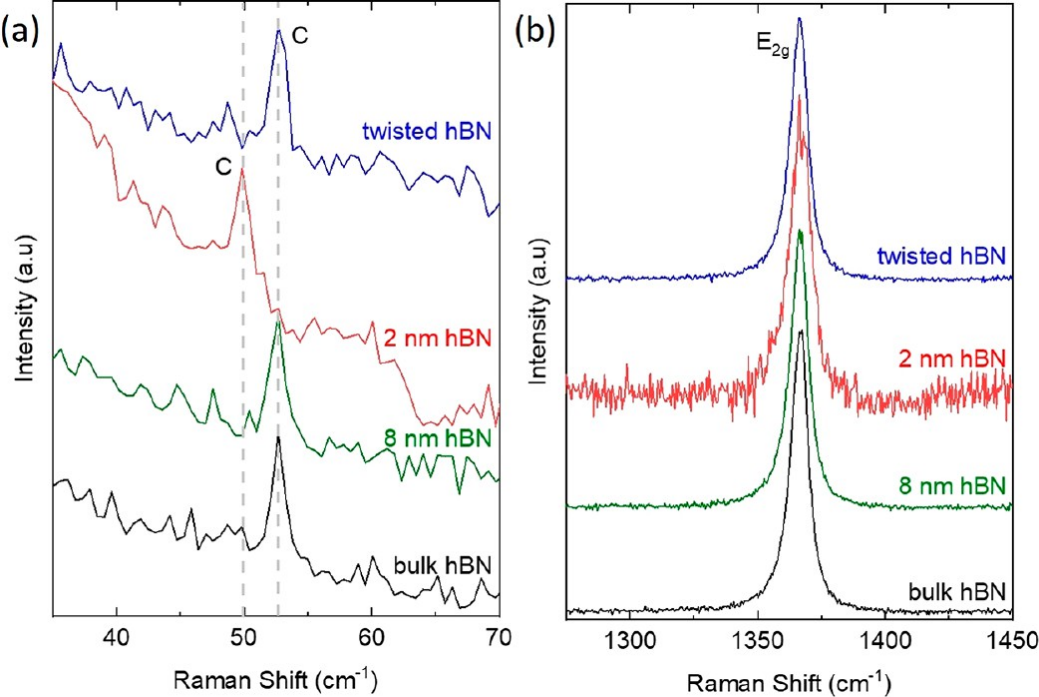
(a) Low and (b) high-frequency Raman spectra of a t-hBN (blue), 2 nm hBN (red), 8 nm hBN (green) on Si + 285 nm SiO_2_ and B-hBN (black).

Figure 7 plots the Raman spectra of the 2 and 8 nm hBN flakes, of the resulting t-hBN and the starting B-hBN on Si + 285 nm SiO_2_. Pos(C) = 52.5 ± 0.14 cm^–1^ for the 8 nm flake, t-hBN and B-hBN, with FWHM(C) = 1 ± 0.2 cm^–1^, whereas Pos(C) = 50.1 ± 0.14 cm^–1^ for the 2 nm flake. Pos(C) can be used to determine *N*, for *N* > 2 as^67,72,73^
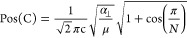
2with *c* the speed of light in cm s ^–1^, μ = 6.9 × 10^–27^ kg Å^–2^ the mass of one layer per unit area and α_⊥_ the interlayer coupling.^67,72,73^

In B-hBN,  ± 0.14 cm^–1^. From this we can derive α_⊥_ = 1.69 × 10^18^ N m^–3^. We then use it in eq 2, and get *N* = 5 ± 1 for the 2 nm thick flake and *N* > 10 for the 8 nm one. Figure 7b gives Pos(E_2g_) = 1366 ± 0.2 cm^–1^ with FWHM(E_2g_) = 8.1 ± 0.2 cm^–1^ for 8 nm, t-hBN, and B-hBN, whereas FWHM(E_2g_) = 9.8 ± 0.2 cm^–1^ for the 2 nm flake. The peak broadening ∼1.7 cm^–1^ in the 2 nm flake can be attributed to strain variations within the laser spot, as thinner flakes conform more closely to the roughness of the underlying SiO_2_. This is also confirmed by the higher RMS roughness of the 2 nm flake (∼0.6 nm) as measured by AFM, compared to ∼ 0.2 nm for the 8 nm flake and t-hBN.

### Phase and KPFM Maps

**Figure 8:**
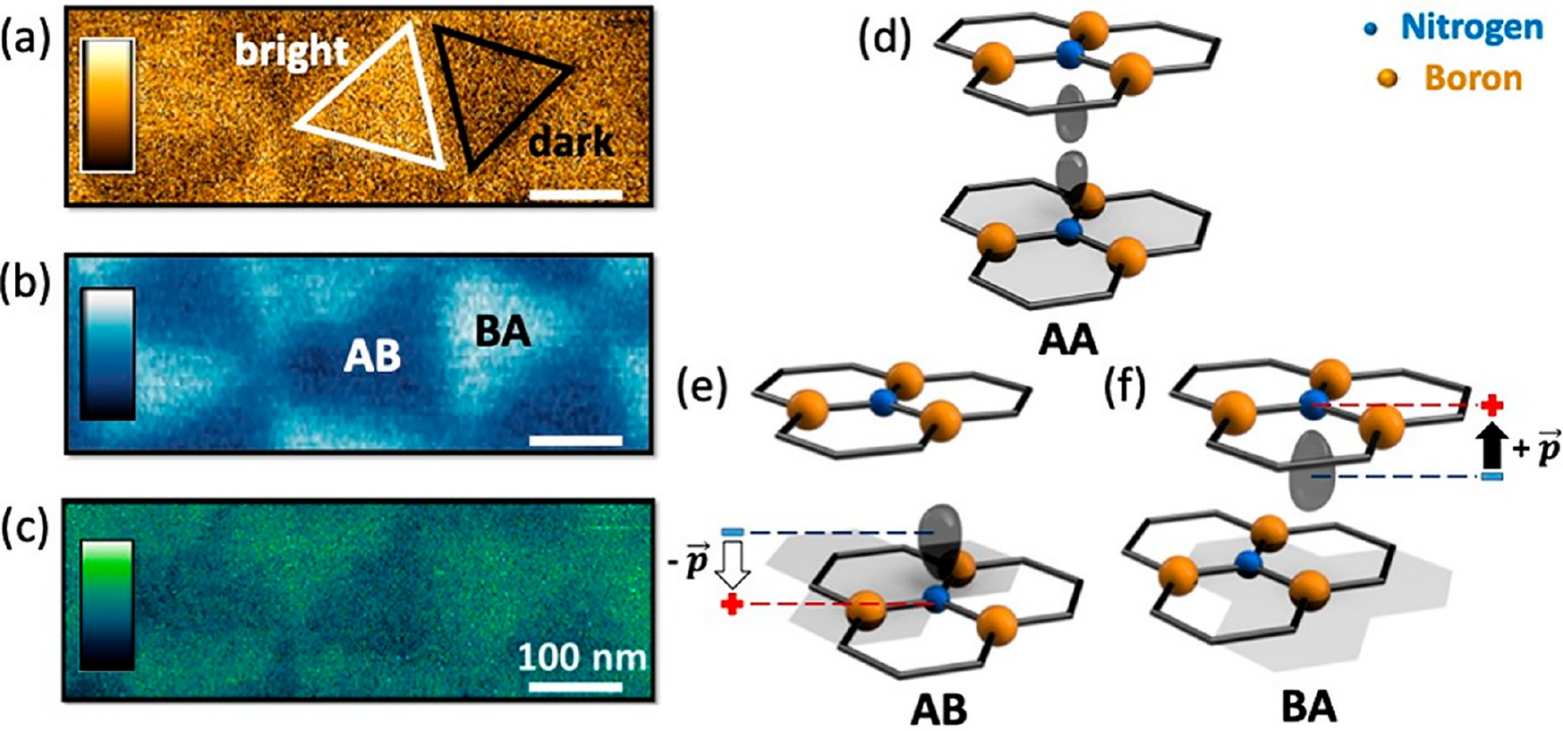
(a) AFM phase image revealing bright and dark triangular moiré phase domains. Color scale: from 101° to 104°. (b) KPFM image acquired from the same region as (a), showing a moiré superlattice, characterized by AB and BA stacking domains. Color scale: from −60 to +60 mV. Image processing: 11th polynomial background removal. (c) Dissipation energy map obtained in the same region as (a) and (b), providing a moiré pattern. Color scale: from 4.7 to 6.4 eV. (d) Schematic of adjacent AA stacked hBN layers characterized by a zero net polarization. (e) AB stacking configuration where a negative electric dipole emerges. (f) BA stacking with a positive electric dipole. All the representations of d–f are not in scale. Imaging parameters for (a,c): *A*_0_ = 16.3 nm, *A* = 15.7 nm, free phase ∼88.2°. Cantilever: 240AC-NG (OPUS, *k* ∼ 2 N·m^–1^). Imaging parameters for (b): *A*_0_ ∼ 25 nm, *A* ∼ 10 nm, lift height = 3 nm, drive voltage = 1 V. Cantilever: ASYELEC.01-R2 (Asylum Research, *k* ∼ 2.8 N·m^–1^).

Figure 8a plots an AFM phase image showing the same moiré superlattice of Figure 1d. Bright and dark regions are highlighted in order to compare with the corresponding KPFM image of Figure 8b. As reported for KPFM measurements (performed positively biasing the AFM tip) on ferroelectric domains,^74,75^ a higher (lower) surface potential corresponds to an upward (downward) polarization, a feature of BA (AB) stacking domains in t-hBN.^22^ Hence, bright (dark) phase domains (defined following the Asylum Research convention, see ref (41)) correspond to AB (BA) regions.

This interpretation of the origin of the energy dissipation map contrast is also in agreement with such domain identification. Figure 8d–f sketch the structure of AA, AB, BA stacking domains. These different alignments are labeled as in refs (20, 22, 76, 77). Due to a symmetric charge distribution of the nitrogen (N) *2p*_*z*_ orbitals, AA has a zero net electric dipole (Figure 8d). The AB configuration (Figure 8e), instead, shows the distortion of the *2p*_*z*_ orbital of the N atom due to its higher electronegativity,^22^ resulting in a downward oriented electric dipole closer to the N atom itself. Figure 8f reports BA stacking, characterized by an electric dipole pointing upward.

Figure 8c shows the dissipation map corresponding to Figure 8a,b. By direct comparison, the BA stacking domain can be addressed as the most dissipative. An interpretation of this can be provided based on the AB electric dipole being deeper in the material than the corresponding BA dipole (as shown in Figure 8e,f). Consequently, the vdW force (inversely related to the tip–sample distance) is larger when the tip is probing a BA domain, thus leading to a higher dissipation (see eq 1).

### KPFM Maps of a t-hBN (2.0 nm/8.0 nm, θ_twist_ ∼ 0.0°)

**Figure 9:**
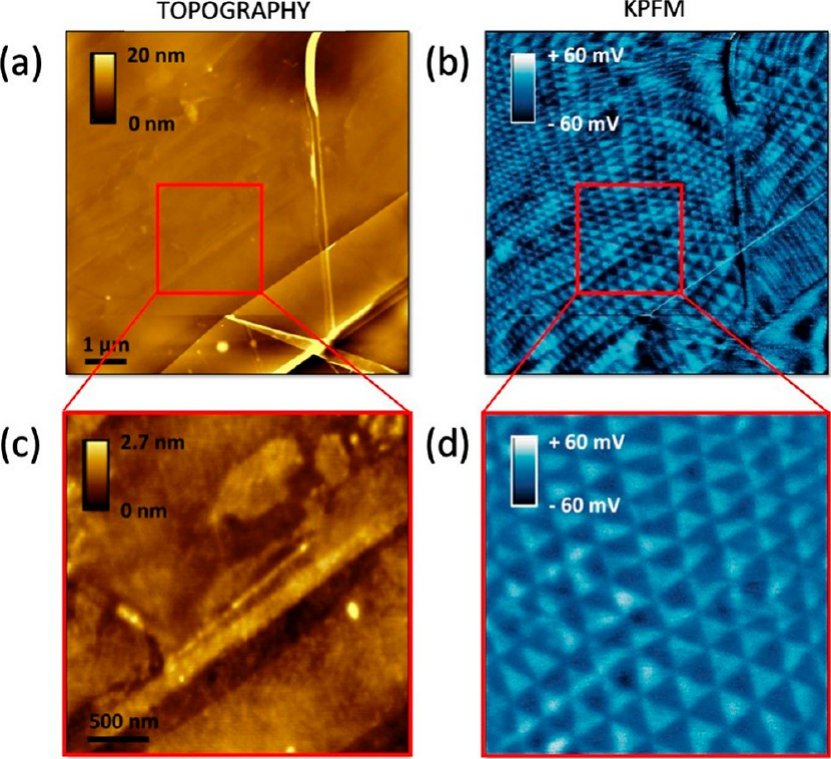
(a,c) Topography, (b,d) KPFM maps. (c,d) Zoom of (a,b). Cantilever: ASYELEC.01-R2 (Asylum Research, *k* ∼ 2.8 N·m^–1^). Imaging parameters: *A*_0_ ∼ 25 nm, *A* ∼ 10 nm, lift height = 3 nm, drive voltage = 1 V. KPFM image processing: flattening, line correction, 11th order polynomial.

Figure 9 plots the topographical (Figure 9a,c) and corresponding KPFM images (Figure 9b,d) of the t-hBN presented in the main text. While the topography is not showing any moiré pattern, the KPFM images have the same triangular shapes as the phase image of Figures 1d, 8a.

### Large Scan Area

**Figure 10:**
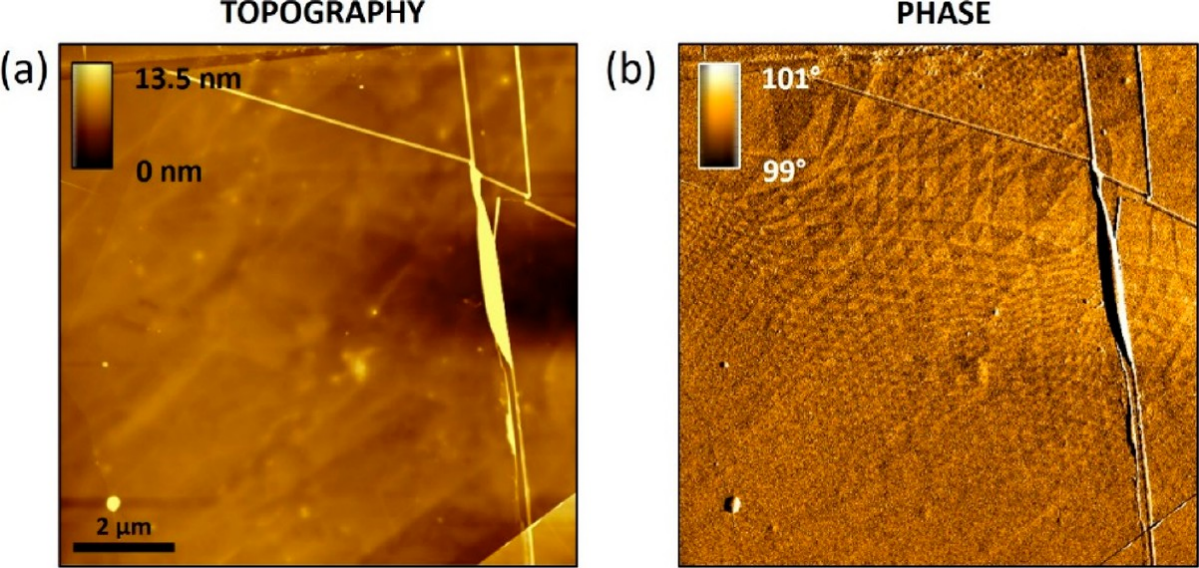
(a) AFM tapping mode topography and (b) corresponding phase image of a 8 μm × 8 μm scan area for t-hBN. A moiré pattern is visualized only in the phase image. Cantilever: Scanasyst fluid (Bruker, *k* ∼ 0.7 N·m^–1^). Imaging parameters: *A*_0_ ∼ 7.2 nm, *A* ∼ 7 nm, free phase ∼82°.

Figure 10 is the topography (Figure 10a) and the corresponding phase image (Figure 10b) obtained for a t-hBN (2 nm/8 nm, θ_twist_ ∼ 0°). The phase image shows a moiré pattern over the whole 8 μm × 8 μm scan size.

### AFM of t-hBN (0.8 nm/5.7 nm, θ_twist_ = 0.2°)

**Figure 11:**
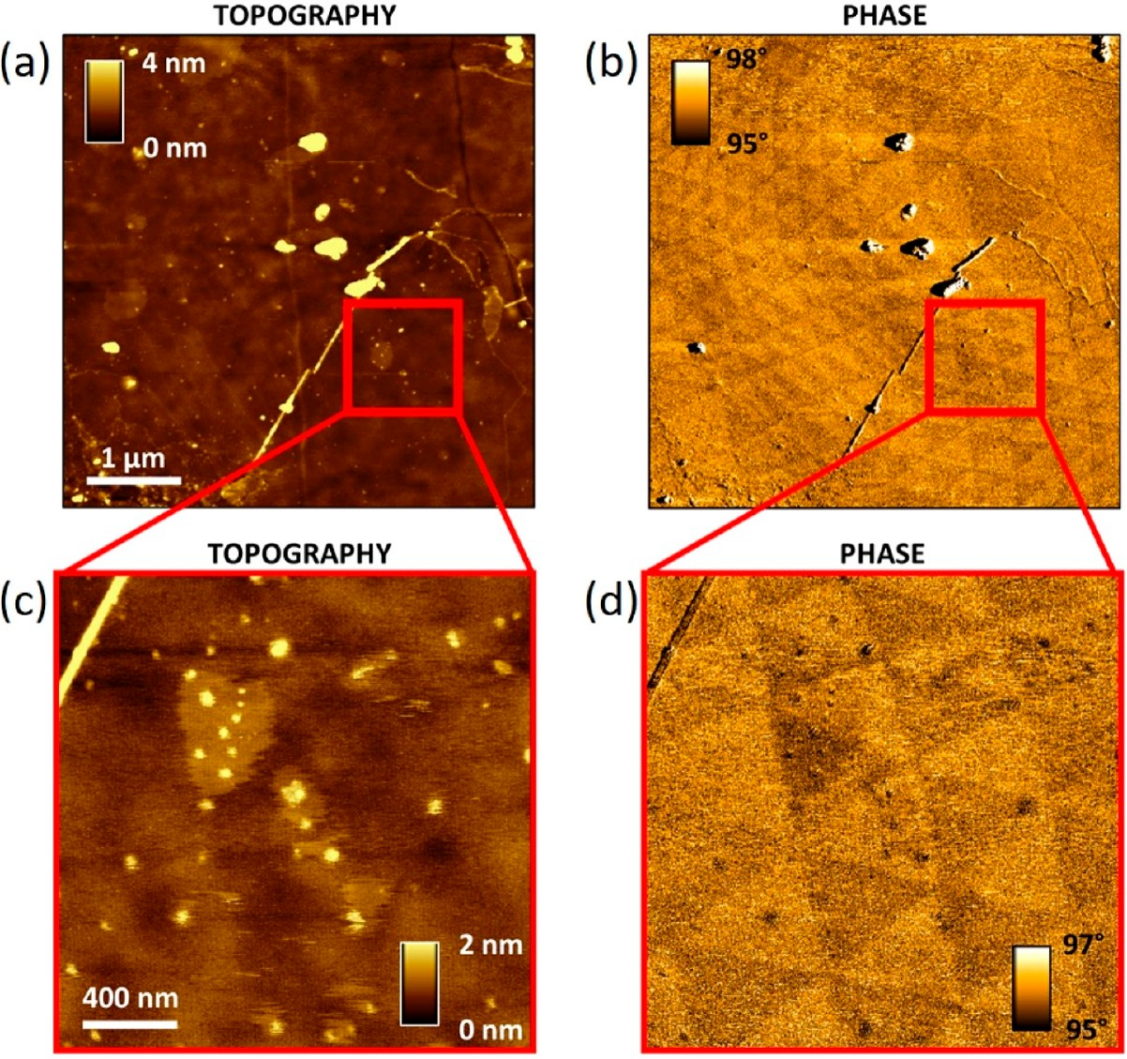
(a,c) AFM tapping mode topography and (b,d) corresponding phase images of a t-hBN (0.8 nm/5.7 nm, θ_twist_ = 0.2°). (c,d) are zooms of (a,b). Despite no contrast in the topography maps, a moiré superlattice is seen in the phase channels. Cantilever: Scanasyst fluid (Bruker, *k* ∼ 0.7 N·m^–1^). Imaging parameters: *A*_0_ ∼ 9.5 nm, *A* ∼ 9 nm, free phase ∼86°.

Figure 11 reports tapping mode AFM topography and phase maps of a t-hBN with different top and bottom layers’ thickness and θ_twist_ (0.8 nm/5.7 nm and 0.2°, respectively) than the one discussed in the main text. While the topography maps (Figure 11a,c) do not show any relevant feature, in the phase images a moiré pattern can be seen.

### Dissipation Maps vs *A*/*A*_0_

**Figure 12:**
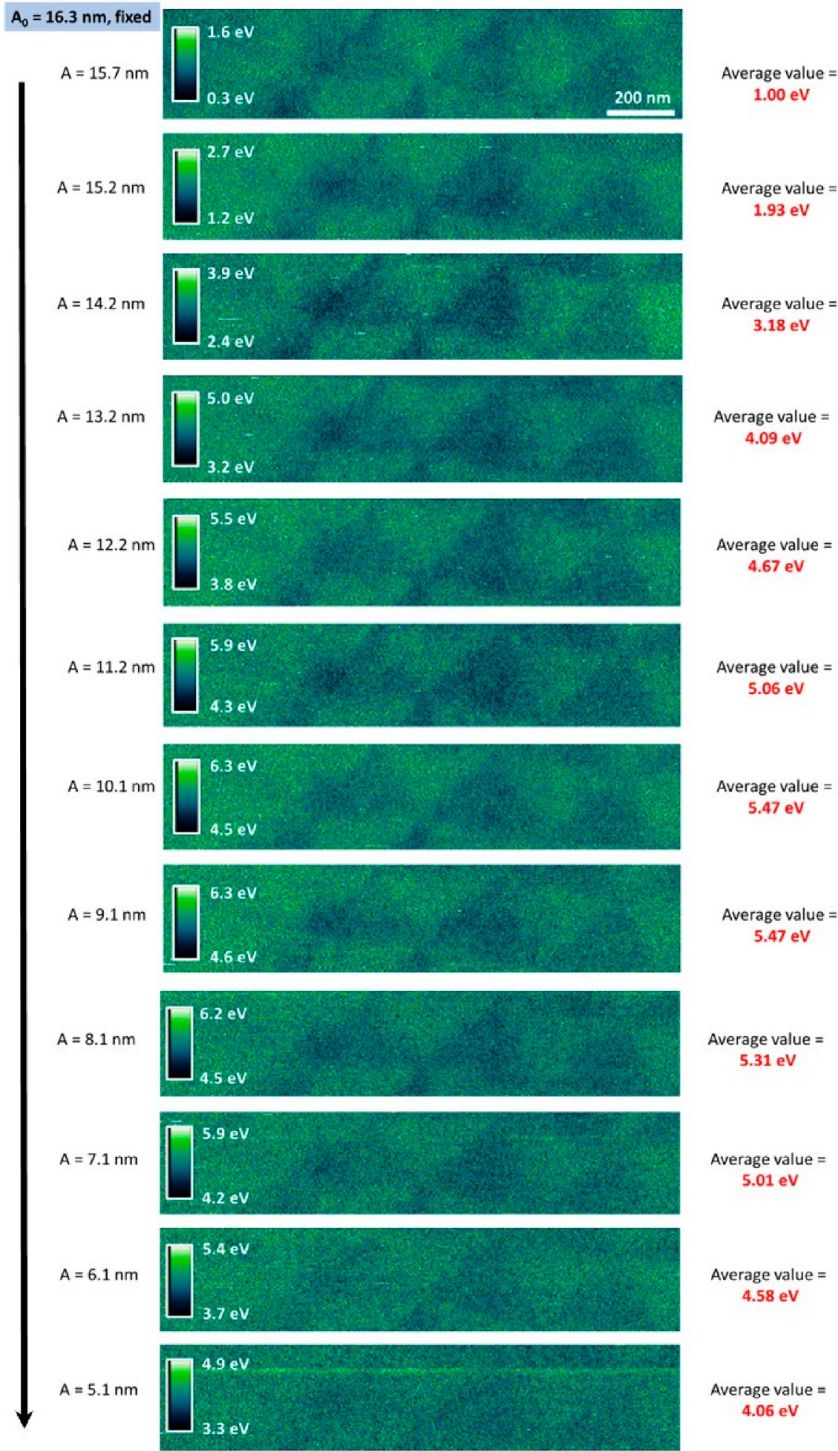
Dissipation maps scanning the same top hBN region at different *A*/*A*_0_. The different *A* are reported, for each image, on the left. *A*_0_ = 16.3 nm. The average dissipation values (reported on the right) are calculated from the corresponding phase images using Gwyddion^40^ following eq 1. Additional imaging parameter: *Q* = 135, scanning frequency = 0.5 Hz, total acquisition time ∼2 h. Cantilever: 240AC-NG (OPUS, *k* ∼ 2 N·m^–1^).

The data of Figure 12 allow us to derive the characteristic curve of Figure 3a. We do not observe flips in the contrast. This is in accordance with the interpretation we provide of the effect of AB and BA stacking. The dipoles of AB and BA sites have different distances from the surface, being the hBN interlayer distance ∼3 Å. This gap is constant whatever the scanning parameters are. The strength of the interlayer dipoles is constant and independent of the scanning parameters. According to these observations, no flip of the contrast should be expected.

Eq 1 can be rewritten as:^26,28^
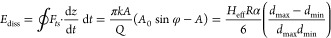
*E*_diss_ depends on the maximum and minimum distance (*d*_max_ and *d*_min_) of the tip from the considered interlayers dipoles.^26^ Therefore, a thicker top-hBN will necessarily increase *d*_max_ and *d*_min_, decreasing *E*_diss_. The thickness of the top layer can affect the formation of the domains itself.^20^ Such effect would complicate the possibility to set a reference for experimentally deriving the trend of the dissipation energy with respect to the increasing distance due to a thicker top layer.

The dissipated energy does not only depend on the tip–sample distance, but also on the hysteresis coefficient α. The physical origin of this parameter is not unique, since several phenomena can contribute to increase the adhesion in the withdraw curves. In ref (78), an extensive list of possible processes is reported. Among them: formation and rupture of chemical bonds between tip and sample, atom reorientation and dislocation, local rearrangement and displacement of atoms. Likely, this would increase the uncertainty in measurements performed on different samples. The only quantitative comparison possible is then between different domains (AB and BA) of the same sample, with the same AFM cantilever, in the same environmental conditions.

### Force–Distance Curves on Both AB/BA Stacking Domains

See Figure 13.

**Figure 13:**
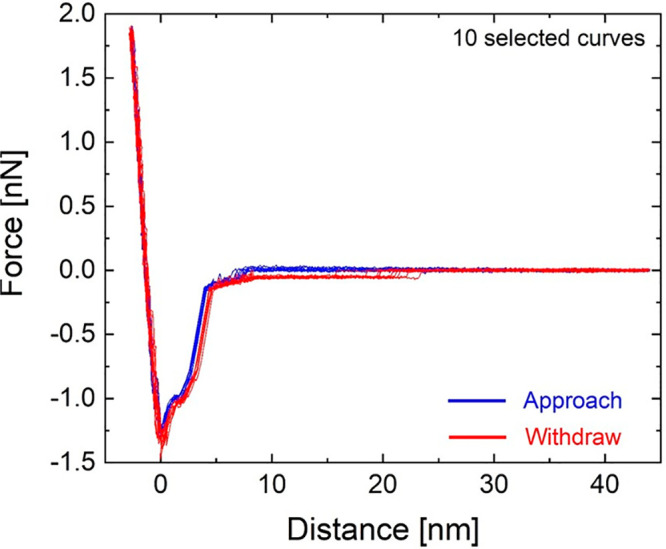
Ten *F*–*d* curves on AB and BA domains showing the same general behavior of Figure 3d, characterized by two different hysteresis regimes: vdW and capillary. *d*_off_ and *d*_on_ are not the same for all curves.

### VdW Hysteresis Description

Long-range dissipative forces act upon the tip in the noncontact attractive regime, and are typically represented by a vdW-like distance-dependent expression^26,78^
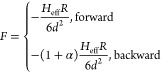
3

In eq 3, the (effective) Hamaker constant, *H*_eff_, represents the magnitude of the vdW interaction between an AFM tip with radius *R* and the sample at a distance *d*.^26,78^ α ≥ 0 distinguishes between forward and backward movements of the tip with respect to the sample during one oscillation.^78^ If the two tip–sample regimes are equal in magnitude (α = 0), a conservative interaction arises providing no dissipation. The existence of a magnitude difference (α > 0), instead, yields a dissipation ∝ α.^28^*H*_eff_ in eq 3 corresponds to an *effective* parameter, taking into account all 3 main interactions between tip and sample-substrate. Since thicknesses are in the few nm range, *H*_eff_ can be identified for tip-ambient-top hBN, tip-ambient-bottom hBN, and tip-ambient-substrate systems.^79,80^

### VdW and Capillary Dissipation Energies

See Figure 14.

**Figure 14:**
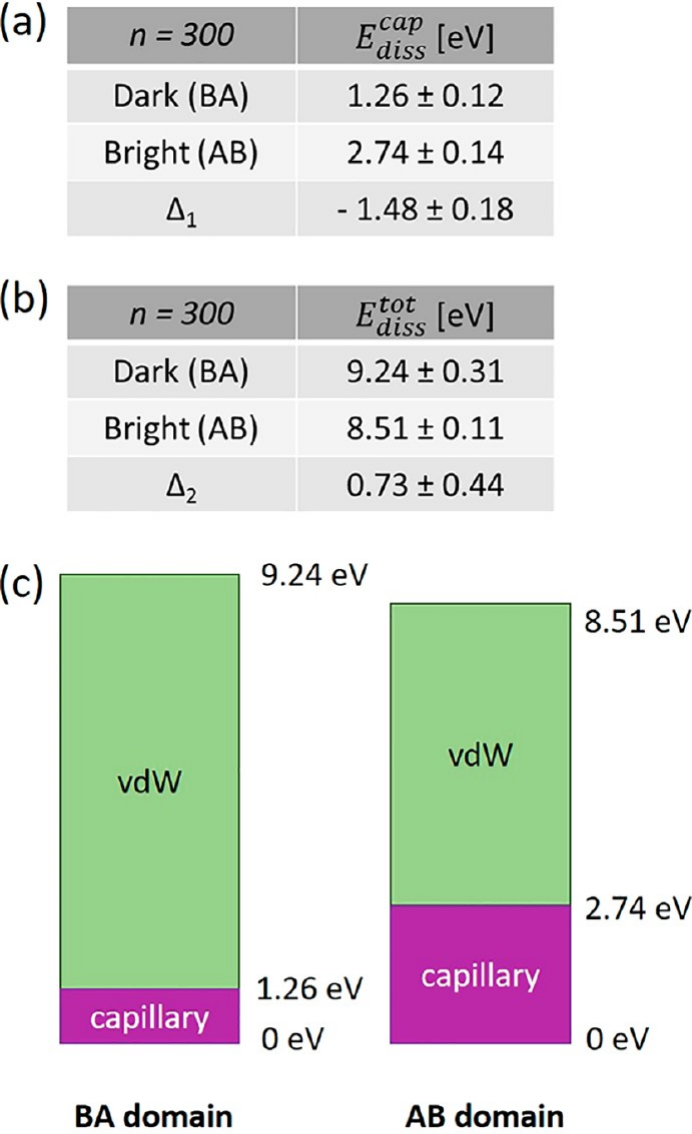
Summary of dissipation energies referred to the 300 *F*–*d* curves of Figure 3d. (a) Table showing, for dark (BA) and bright (AB) stacking domains, the average capillary dissipation energy *E*_diss_^cap^ and the related energy difference Δ_1_. *E*_diss_^cap^ is calculated through a Python code capable of evaluating the area between approach and withdraw curves restricted to the capillary hysteresis regime of Figure 3d. (b) Table showing the average total dissipation energy *E*_diss_^tot^, sum of the capillary and the vdW nonconservative contributions (the vdW energies are in Table 1). The related energy difference Δ_2_ is also reported. (c) Schematic of the main energies considered in (a,b) for dark (BA) and bright (AB) domains. The vdW dissipation contribution is in green and the capillary dissipation in violet. The main energy values are also shown.

### Topography and Phase Maps in Different Areas

**Figure 15:**
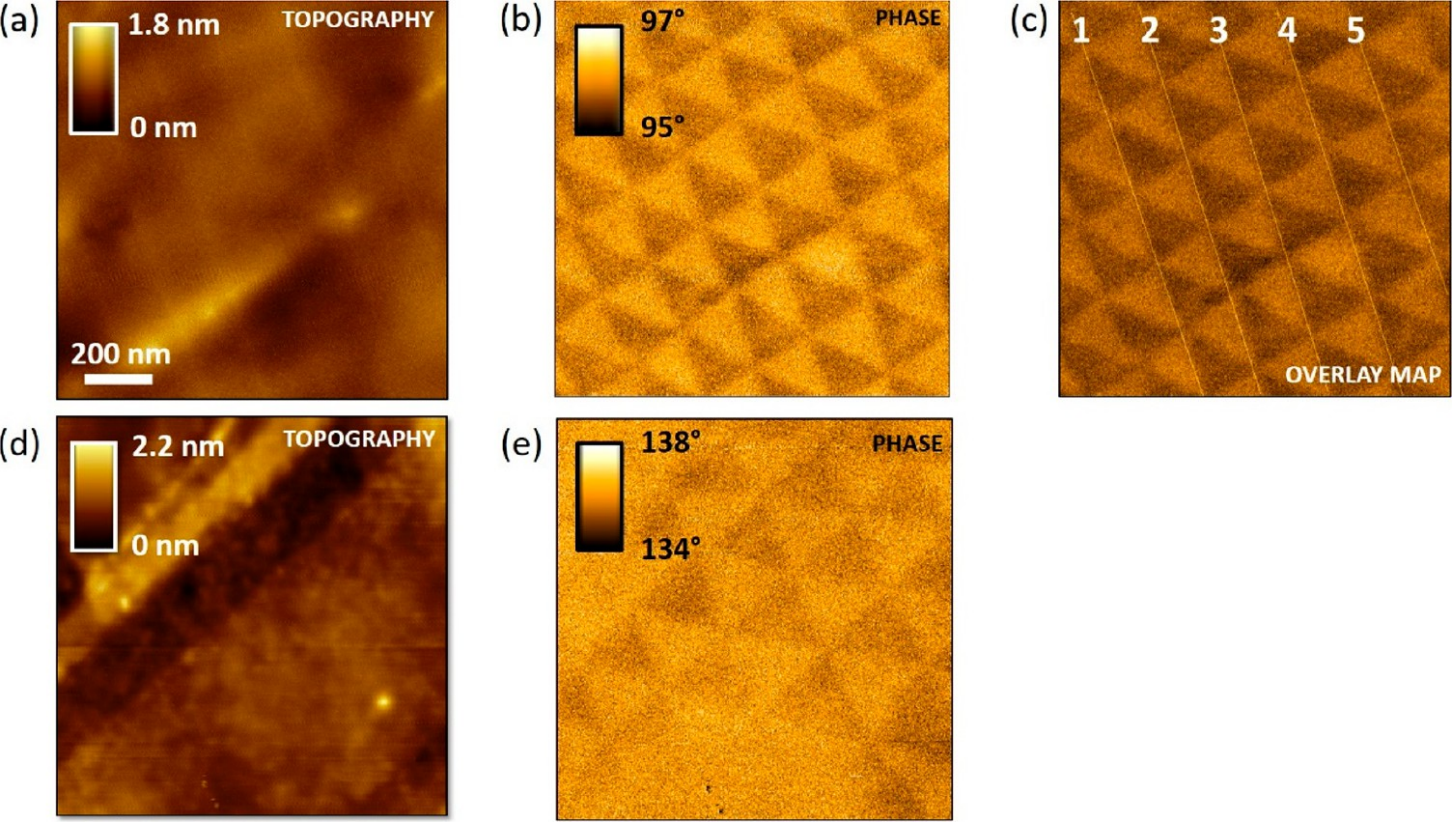
(a) AFM topography and (b) corresponding phase image (equal to Figure 1c,d) of a t-hBN (2.0 nm/8.0 nm, θ_twist_ ∼ 0.0°) sample. The topography channel shows several straight lines, while a full moiré pattern can be seen in the phase channel. (c) Five main lines in the topography channel in panel (a) overlaid onto the phase channel (b). A clear correspondence between their position and the moiré superlattice (in one direction) is visible. (d) AFM topography and (e) corresponding phase image of a different region (same sample) with respect to case (a) and (b).

In Figure 1c,d the topography is characterized by a flat morphology plus several straight lines. These are overlaid onto the panel (b) phase map in Figure 15c, providing a direct visualization of their correlation with the moiré pattern (at least in one direction). These lines could be either a real local deformation (∼1 Å), induced by the underneath moiré superlattice, or an apparent topography, following from a different vdW interaction. When imaging in tapping mode nm-scale samples, such as nanoparticles, DNA or hBN flakes, the vdW force between tip and underneath substrate can influence an apparent AFM-height. We do not always observe these additional lines. As shown in Figure 15e, while the phase channel has a moiré superlattice, the corresponding topography does not have any moiré-related feature. Similar considerations apply to Figure 11 (different sample), where the topography channel does not show any feature immediately related to the probed moiré superlattice.

### AFM Phase Imaging of *t*-WSe_2_

**Figure 16:**
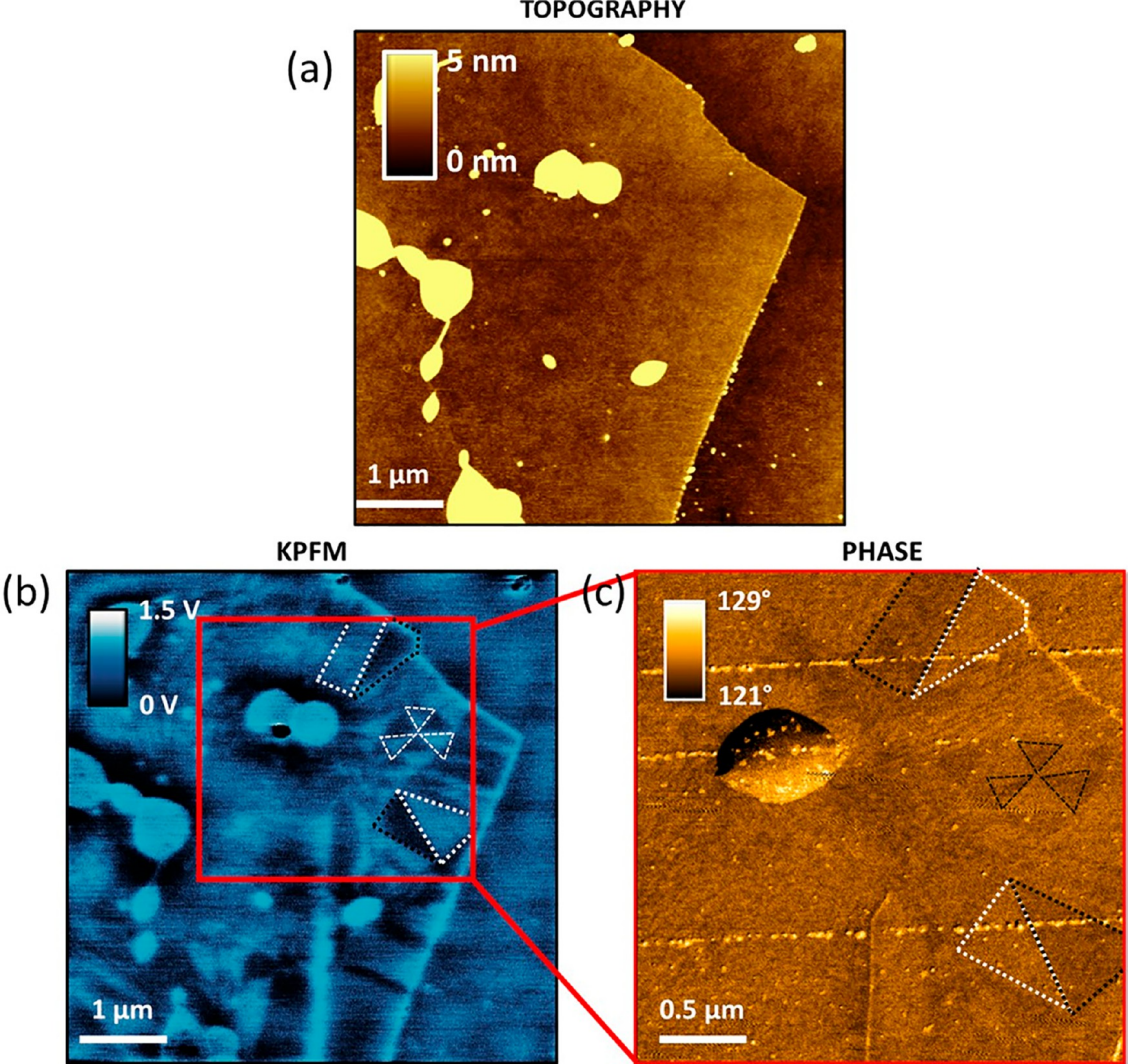
(a) Tapping mode AFM topography of a WSe_2_/WSe_2_ (2.1 nm/2.9 nm, θ_twist_ ∼ 0°). (b) Corresponding KPFM image addressing a moiré pattern highlighted by black and white dash lines. (c) Tapping mode AFM phase image of the red square zoomed area (see panel b): the same moiré contrast visualized by KPFM can be distinguished. Cantilever KPFM: ASYELEC.01-R2 (Asylum Research, *k* ∼ 2.8 N·m^–1^). Imaging parameters: *A*_0_ ∼ 25 nm, *A* ∼ 10 nm, lift height = 3 nm, drive voltage = 1 V. KPFM image processing: flattening, line correction, 11th order polynomial. The z-scale is logarithmic to enhance the contrast. Cantilever phase-imaging: Scanasyst air (Bruker, *k* ∼ 0.7 N·m^–1^). Imaging parameters: *A*_0_ ∼ 5 nm, *A* ∼ 4 nm, free phase ∼88°.

Figure 16 plots tapping mode AFM topography, KPFM and phase images of 1L-WSe_2_/1L-WSe_2_ (2.1 nm/2.9 nm, θ_twist_ ∼ 0°) on Si + 285 nm SiO_2_. While the morphology (Figure 16a) does not provide any moiré contrast, the KPFM image has some triangular domains highlighted by black and white dash lines. The same moiré KPFM domains are obtained by tapping mode AFM phase-imaging scanning the zoomed red square reported in (Figure 16b).

### Force–Distance Curves on AB/BA Domains of t-hBN (0.8 nm/5.7 nm, θ_twist_ = 0.2°) Sample

**Figure 17:**
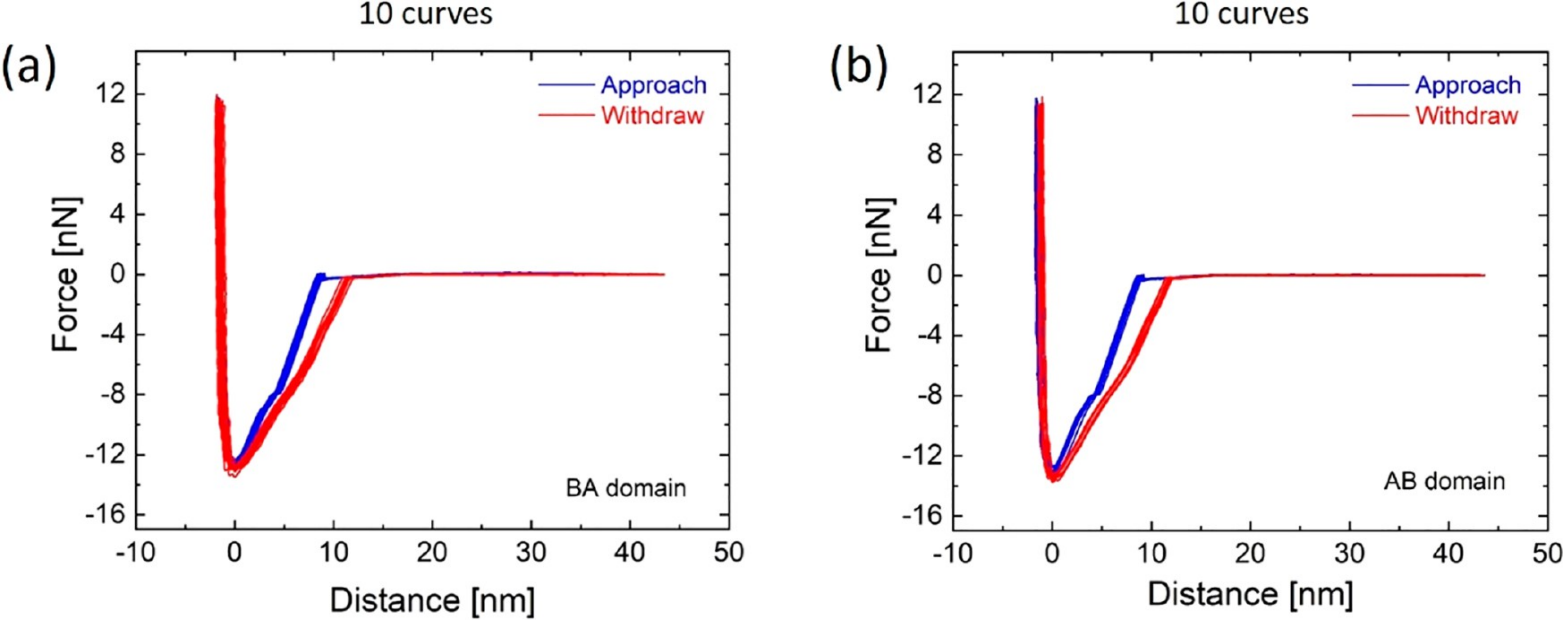
(a) 10 selected *F*–*d* curves (out of 300) measured at the center of a BA domain. (b) 10 selected *F*–*d* curves (out of 300) measured at center of an AB domain. *k* = 2.12 N·m^–1^.

Figure 17 plots 10 selected *F*–*d* curves (out of 300) measured on the center of both BA (Figure 17a) and AB (Figure 17b) domains for the t-hBN (0.8 nm/5.7 nm, θ_twist_ = 0.2°) sample of Figure 11. In both cases, approach and withdraw curves do not overlap, giving rise to a hysteresis. The corresponding dissipated energy can be obtained calculating the area in between them. Notably, we get a higher average dissipation for BA domains (∼170 eV) than for AB regions (∼162 eV). The different force values with respect to Figure 13 are due to the use of cantilevers with different stiffness: 0.75 N·m^–1^ in Figure 13; 2.12 N·m^–1^ for Figure 17.
